# Clinical, physical and lifestyle variables and relationship with cognition and mood in aging: a cross-sectional analysis of distinct educational groups

**DOI:** 10.3389/fnagi.2014.00021

**Published:** 2014-02-24

**Authors:** Nadine C. Santos, Patrício S. Costa, Pedro Cunha, Carlos Portugal-Nunes, Liliana Amorim, Jorge Cotter, João J. Cerqueira, Joana A. Palha, Nuno Sousa

**Affiliations:** ^1^Life and Health Sciences Research Institute (ICVS), School of Health Sciences, University of MinhoBraga, Portugal; ^2^ICVS/3B's, PT Government Associate LaboratoryBraga/Guimarães, Portugal; ^3^Clinical Academic Center – BragaBraga, Portugal; ^4^Centro Hospitalar do Alto Ave – EPEGuimarães, Portugal

**Keywords:** neurocognitive/neuropsychological assessment, structural equation modeling, cognition, hierarchical regression, stepwise regression

## Abstract

It is relevant to unravel the factors that may mediate the cognitive decline observed during aging. Previous reports indicate that education has a positive influence on cognitive performance, while age, female gender and, especially, depressed mood were associated with poorer performances across multiple cognitive dimensions (memory and general executive function). Herein, the present study aimed to characterize the cognitive performance of community-dwelling individuals within distinct educational groups categorized by the number of completed formal school years: “less than 4,” “4, completed primary education,” and “more than 4.” Participants (*n* = 1051) were randomly selected from local health registries and representative of the Portuguese population for age and gender. Neurocognitive and clinical assessments were conducted in local health care centers. Structural equation modeling was used to derive a cognitive score, and hierarchical linear regressions were conducted for each educational group. Education, age and depressed mood were significant variables in directly explaining the obtained cognitive score, while gender was found to be an indirect variable. In all educational groups, mood was the most significant factor with effect on cognitive performance. Specifically, a depressed mood led to lower cognitive performance. The clinical disease indices cardiac and stroke associated with a more negative mood, while moderate increases in BMI, alcohol consumption and physical activity associated positively with improved mood and thus benefitted cognitive performance. Results warrant further research on the cause-effect (longitudinal) relationship between clinical indices of disease and risk factors and mood and cognition throughout aging.

## Introduction

The overall cognitive decline observed during aging deserves a two-fold consideration: the study of common factors that may explain the overall population shift and of those that may differentially affect individual performance [review, (Salthouse, 2010)]. These can include lifestyle parameters which may be of particular relevance due to their modifiable nature and possible effect on attenuating, preventing or even reversing cognitive decline (Fratiglioni et al., 2004; Simons et al., 2006; Kalaria et al., 2008; Scarmeas et al., 2009; Yamamoto et al., 2009; Shubert et al., 2010; Snowden et al., 2011; Miller et al., 2012). Multiple aspects comprise lifestyle, including physical and social activity and dietary/nutrition habits, which combined and/or individually can influence cognition. Favorable lifestyle interventions, such as nutrition education and/or increasing physical activity, have been associated with cognitive improvements (Yamamoto et al., 2009), lowered risk of dementia (Simons et al., 2006), partial rescue of some cognitive deficits (Shubert et al., 2010; Snowden et al., 2011; Miller et al., 2012), reduced risk for the development of Alzheimer's disease (Scarmeas et al., 2009), and an ameliorating role on pathologies such as metabolic syndrome (including on its individual disease components) (Alberti et al., 2005; Dik et al., 2007).

Additionally, certain clinical indices of disease and risk factors have been associated with depressed mood and cognition (van Gool et al., 2003, 2007). For instance, older individuals with metabolic syndrome also exhibit higher prevalence of depressive symptoms (Viscogliosi et al., 2013). Also, while chronic conditions such as diabetes mellitus, stroke and/or transient ischemic attack (TIA) may not necessarily contribute to cognitive decline over time, preventing/diagnosis of metabolic and cardiovascular diseases may be essential to cognitively healthy aging (Köhler et al., 2012). Studies also indicate for a complex association between body mass index (BMI) and cognition. While on one hand, obesity appears to be associated with lowered cognitive performance (Elias et al., 2005; Fergenbaum et al., 2009), on the other, a BMI greater than 25.0 kg/m^2^ has been associated with a cognitive protective effect among aging individuals (Atti et al., 2008), with a BMI lower than 25.0 coinciding with a worse cognitive status in older demented individuals (Coin et al., 2012). Furthermore, depressive mood has also been associated with cognitive performance, where a more negative mood may act together with educational level to promote transition to a more negative cognitive status (Minicuci et al., 2005; Santos et al., 2013).

Finally, socio-demographic variables are also of key interest (Paulo et al., 2011; Santos et al., 2013). In particular, education is considered a major factor explaining cognitive trajectories throughout aging (Ardila et al., 2000; Wilson et al., 2009). In this context, Portugal is a particularly interesting population-based case study given the similarity of the current and forecasted educational attainment of the Portuguese population to that worldwide. That is, albeit its aging population being less educated than those in Western European and North-American countries, it is similar to most other more newly developed and/or developing countries. Presently, primary education mainly characterizes the middle-aged and older Portuguese population (that is, completion of the 4 school years that comprise basic grade school), with low percent scores having completed preparatory (grades 5–8), secondary (grades 9–12) and tertiary (post-secondary, college/university) levels. However, recent census data indicates that the number of young individuals with tertiary education has already doubled in the last decade (INE, 2011). Multi-database projection analysis on levels of educational attainment points toward a similar scenario in most countries (Lutz and Kc, 2011). In the present study, the Switchbox Consortium (http://www.switchbox-online.eu/) explored the hierarchical and combined influence of mood, clinical, physical and lifestyle variables across distinct educational groups, on age-related cognitive performance.

## Materials and methods

### Ethics statement

The study was conducted in accordance with the Declaration of Helsinki (59th Amendment), and was approved by national and local ethics committees. Potential participants were explained the study goals and the neurocognitive/psychological and clinical assessments. All volunteers provided informed consent.

### Sample characteristics

Participants (*n* = 1051, final sample size after exclusion criteria; males and females, 50+ years of age) were randomly selected from the Guimarães and Vizela local area health authority registries. Portuguese citizens are registered in local health centers since birth and are automatically assigned a family and general practitioner (GP). The sample is part of a larger cohort (*n* = 3038, males and females 18–97 years of age, from an original *n* = 4000; drop-out rate for the age group 50 or more years of age, 27.8%). For age and gender, the distribution of this database differs in less than 2% of that of the distribution for the Portuguese population estimated by the Portuguese authority on statistics, the “Instituto Nacional de Estatística” (INE, 2011). The primary exclusion criteria included inability to understand informed consent, participant choice to withdraw from the study, incapacity and/or inability to attend the clinical and neuropsychological assessment session(s), dementia and/or diagnosed neuropsychiatric and/or neurodegenerative disorder (medical records). A team of experienced clinicians performed a standardized clinical interview (with self-report by the participants) that also addressed current medication and allowed to further detect and exclude disorders of the central nervous system (epilepsy and neurodegenerative disorders) as well as overt thyroid pathology.

### Clinical, physical, and lifestyle variables

General health aspects considered included clinical history of: coronary/cardiac disease or insufficiency (including coronary bypass, peripheral vascular disease, cardiac insufficiency, myocardial infarction, coronary disease, and arrhythmia), diabetes (including diabetes mellitus type I and II and diabetic retinopathy), stroke (including ischemic stroke, hemorrhagic stroke, and TIA), dyslipidemia and hypertension. Measures are self-reported and confirmed from medical records. Nonetheless, without throughout disease-directed exams at the time of inclusion in the study, undiagnosed pathologies and/or more recent events cannot be fully ruled out. For instance, particularly in older individuals, smaller strokes may go undiagnosed without updated structural imaging studies, possibly also missing microangiopathy and white matter loss, or even a fraction of larger territorial infarctions.

Physical measures included weight (Kg), height (m) and abdominal perimeter (cm). Body mass index (BMI, Kg/m^2^) was categorized as underweight, normal, overweight and obese (respectively, BMI: [0–18.5], [18.6–24.9], [25.0–29.9], and [30.0+]) (WHO, 2008). For statistical procedures the underweight and normal categories were combined due to the small sample size for underweight (*n* = 5). Metabolic risk was categorized from normal, to increased and substantially increased (respectively, abdominal perimeter (cm): females, [0–80.0], [80.1–88.0] and [88.1+]; males, [0–94.0], [94.1–102.0], and [102.1+]) (WHO, 2008). For lifestyle, alcohol consumption (none, 50 or less and more than 50 gr/day), physical activity status (none, less than 3, and over 3 times per week) and smoking habits (non-smoker, former smoker, and smoker) were considered. For drinking habits, alcohol consumption was calculated and recorded as total gr/day. Based on the most commercially available, the following equivalencies were used: 44 gr of alcohol/100 mL spirits/hard liquor (“aqua vitae”); 35 gr of alcohol/100 mL gin, rum, whisky and brandy; 28 gr of alcohol/100 mL sweet/dessert type liqueur; 3.7 gr of alcohol/100 mL beer; 9.6 gr of alcohol/100 mL red and white wine; 8.4 gr of alcohol/100 mL “vinho verde.” Physical activity included any planned activities (e.g., walking, jogging, swimming) that comprised a continuous 30-min effort (which could range from light, to moderate and vigorous) above the everyday living activities such as the case of regular short walks to the grocery store. Activity quantity rather than intensity was considered due to the mixed clinical profiles and age range of the study population. Basal heart rate was not registered to assess effort level. Alcohol consumption, smoking habits and alcohol consumption were self-reported by the participants during the clinical interview.

### Neurocognitive evaluation

A team of trained psychologists conducted the neurocognitive/psychological assessments. Tests were selected to provide mood and cognitive (general cognitive status and executive and memory functions) profiles, as previously reported (Paulo et al., 2011; Santos et al., 2013). The Geriatric Depression Scale (GDS, long-version) (Yesavage et al., 1983) was used to assess depressive mood. The Digit Span Test (subtest of the Wechsler adult intelligence test WAIS III, 1997; parameters: digit span forward and backward) (Wechsler, 1997) as a measure of short-term verbal working memory (immediate retention) and attention (Della Sala et al., 1995; Adams et al., 1996; Wechsler, 1997; Spreen and Strauss, 1998). The Digits Span Test is considered to require an executive-level function [so called “episodic buffer” (Baddeley, 2000)], and it consists of a list of numbers that the participant is asked to repeat back in the correct order (Digits forward) immediately after presentation. Backward memory span (Digits backward) is a variation that involves recalling items in the reverse order. Digits forward is generally regarded as more of an auditory immediate working memory probe (“buffer” capacity). On the other hand, the digit backwards span involves manipulation of content internally; as such, it is considered the more challenging probe and where there is at least some executive load. The Buschke Selective Reminding Test was used as a multiple trial verbal learning and memory test [SRT, parameters: consistent long term retrieval (CLTR), long term storage (LTS), delayed recall and intrusions] (Buschke et al., 1995). The SRT mainly involves episodic verbal memory. A list of 12 words is read to the participant. In the first trial, the participant is asked to recall as many items as possible. In the subsequent five trials, only the items that were not recalled in the preceding trial are read back to the participant. When the participant recalls a word on two consecutive trials, it is assumed to have entered LTS. Words consistently recalled in all subsequent trials are scored as CLTR, those recalled after 20 min as delayed recall, and incorrectly named words (words that are not part of the 12-word list) are considered intrusions (Buschke et al., 1995). The Stroop Color and Word Test (parameters: words, colors and words/colors) (Strauss et al., 2006) was selected to evaluate response inhibition/cognitive flexibility. The Stroop test is based on the premise of the inhibition of an over-learned response by a competing response. The ability to resist interference is commonly used as an indicator of selective attention, cognitive flexibility and response inhibition and as a tool to assess executive function (Strauss et al., 2006). Verbal/phonetic fluency was assessed using the Controlled Oral Word Association test F-A-S (COWAT-FAS, parameters: admissible and non-admissible) (Lezak et al., 2004). The COWAT utilized consisted of the three letter set of “F,” “A,” and “S.” Participants are given 1 min to name as many words as possible beginning with the first letter F and the procedure is repeated for the remaining letters. Two measures can be calculated: admissible (sum of all correct words named by the participant; meaning, existing words the participant correctly named that started with the letter stated), and non-admissible words (sum of all incorrect words named by the participant; including inexistent words, words that did not start with the stated letter and/or repeated words). The Digit Symbol Substitution Test (DSST, subtest of the Wechsler adult intelligence test WAIS III, 1997) (Wechsler, 1997; Strauss et al., 2006) was used as a measure of high-level information processing speed. Finally, global cognitive status was assessed with the Mini-Mental State Examination (MMSE) (Folstein et al., 1975).

The MMSE remains one of the most widely used cognitive mental status-screening instruments (Molloy and Standish, 1997), despite its limitations particularly in sensitivity for subtler cognitive deficit (Brayne and Calloway, 1990; Tombaugh and McIntyre, 1992). More so, the threshold should be adjusted depending on factors such as education (Folstein et al., 1975; Grigoletto et al., 1999; Busch and Chapin, 2008). Here, the following thresholds were used for cognitive impairment: a total MMSE score <17 if individual with ≤4 years of formal school education and/or ≥72 years of age, or a total score of <23 if individual with ≥5 years of formal school education and/or ≤71 years of age (Paulo et al., 2011; Santos et al., 2013). This also follows the validation study for the Portuguese population (Guerreiro et al., 1994). Participants that met the established MMSE threshold criteria (*n* = 51) or that were unable/unwilling to complete the test (*n* = 7) were excluded from further analysis. Of consideration, the exclusion criteria applied prior to inclusion in the study sample (as above described), or an underestimation of cognitive deficit by the MMSE, may have precluded the presence and/or the identification of more cases.

### Statistical analysis

This study aimed to characterize cognitive/mood performance as it related with clinical, physical and general lifestyle-related parameters, in distinct educational groups. Data structuring and analysis followed and expanded on previously reported methodology (Santos et al., 2013). Neurocognitive variable scores were (1) converted into z scores to express all variables in the same scale, and participants with (2) zMMSE values ≤−3 (*n* = 3) were further excluded from the analysis. Next, (3) principal component analysis (PCA) was conducted to reduce information of multiple parameters (here, neurocognitive test variables) into a minimal number of components (that, cognitive/mood “dimensions” or “variable groupings”). From the PCA, a cognitive dimension score was calculated for each individual, allowing for one possible missing value in each dimension. Next, (4) structural equation modeling (SEM) analysis was performed to obtain a derived cognitive score based on the identified cognitive dimensions (measurement model) and to explore causal and correlation links between variables and their effect on cognition (structural model). SEM allows estimating, from measured variables, a variable that is not directly measured (these systematic unmeasured variables may also referred to as factors or latent variables). The derived cognition score was based on the identified neurocognitive/psychological dimensions (PCA), and four predictors with already observed high predictive power (Paulo et al., 2011; Santos et al., 2013) were considered: gender, age, school years (as a non-categorical variable) and zGDS. The model goodness-of-fit was evaluated using the χ^2^ statistics as well as the following indices: comparative fit index (CFI), root mean square error of approximation (RMSEA) and 90% confidence interval (CI) (Hu and Bentler, 1999; Schermelleh-Engel et al., 2003).

Next, to characterize the cognitive performance of community-dwelling individuals within distinct educational groups, the cohort was categorized by number of completed formal school years: “less than 4,” “4, completed primary education,” and “more than 4.” Finally, this was followed by (5) hierarchical linear regression analysis to determine the contribution of the socio-demographic, clinical indices of disease and “lifestyle” variables on neurocognitive/psychological performance. Hierarchical regression is the practice of building successive linear regression models, each adding more predictors. Hierarchical models (also termed, hierarchical linear models) are a type of linear regression models in which the observations fall into hierarchical or completely nested levels and are a type of multilevel models. This statistical approach allows to establish hierarchies of predictors entering the model and to determine the individual contribution of each block of variables as well as contribution when others are also accounted for. The study considered three blocks: age and gender (block 1), clinical indices of disease and GDS (block 2), and lifestyle (block 3) variables. Specifically, the first block included the socio-demographic predictor variables, age and gender (“structural” variables). The second block consisted of the clinical indices of disease (stroke, cardiac pathology, diabetes, dyslipidemia, hypertension) and GDS variables. Lastly, the third block included the “lifestyle indicators” variables: alcohol consumption, physical activity, BMI and metabolic risk. Smoking habits were not considered because 96.6% of the females were non-smokers (therefore, analysis would be biased evaluating the effect of gender and not of smoking habits). Finally, using the same variables, (6) regressions were conducted using the statistical method stepwise for variable entering in the model. The strategy confirms the hierarchical linear regression results, as well as allows to obtain only significant variables in the final model. For both regression procedures, the number of models explored corresponded to the number of dependent variables (that is, number of cognitive/mood dimensions identified from the PCA).

## Results

### Sample characteristics

The cohort was representative of the general Portuguese population with respect to gender (females, *n* = 560 or 53.3%) and age [range: 50–97 years; *M* = 67.2, *SD* = 9.24; age categories: [50–60], 25.4% (females, 52.8%); [60–70], 31.2% (females, 53.7%); [70+], 43.4% (females, 53.3%)]. All participants were community-dwellers and the majority in the medium socio-economic stratum in the Graffar scale (Class III; 61.6%, females 47.3%) and retired (*n* = 763, females 51.8%). A Class III in the Graffar corresponds to middle class (scores between 14 and 17, in a total possible score of 25; higher scores represent higher socio-economic classes) (Graffar, 1956). The literacy rate was 92.2% and the median years of the schooling was 4. Specifically, 34.7% (females 71.0%), 49.4% (females 47.4%) and 15.9% (females 32.9%) of the cohort attended school for [0–3], [4], and ≥5 years, respectively. On socio-demographic measures, Portugal ranks close to the OECD (Organisation for Economic Co-operation and Development; www.oecd.org/) average (OECD, 2012). Table 1 presents the clinical, general lifestyle and physical characterization of the cohort by total and percent number of cases reported for each variable, for males and females.

**Table 1:** **Clinical, general lifestyle and physical characterization of the cohort by valid percent number of cases reported for each variable for males and females**.

	**Percent of cases**	**Gender**	
		**Female**	**Male**
**CLINICAL CHARACTERISTICS**			
Pathology	*n* = 851^a^		
Stroke (%)	9.0	46.8	53.2
Cardiac pathology (%)	17.5	41.6	58.4
Diabetes (%)	24.2	49.5	50.5
Dyslipidemia (%)	67.5	56.4	43.6
Hypertension (%)	71.0	56.8	43.2
Total (%)	189.2	54.6	45.4
**LIFESTYLE AND PHYSICAL CHARACTERISTICS**			
Alcohol consumption (gr/day)	*n* = 1031^b^		
None (%)	29.4	77.9	22.1
50 or less (%)	46.8	55.2	54.8
More than 50 (%)	23.9	17.5	82.5
Total (%)	100.0	52.9	47.1
Physical activity (times per week)	*n* = 1042^b^		
None (%)	64.3	56.0	44.0
Less than 3 (%)	14.8	14.8	14.7
Over 3 (%)	20.9	20.9	20.7
Total (%)	100.0	53.3	46.7
Smoking habits	*n* = 1042^b^		
Non-smoker (%)	70.5	72.9	27.1
Former smoker (%)	22.3	3.9	96.1
Smoker (%)	7.2	13.3	86.7
Total (%)	100.0	53.3	46.7
BMI	*n* = 1007^b^		
Underweight/Normal (%)	23.2	45.7	54.3
Overweight (%)	45.6	47.1	42.9
Obese (%)	31.2	65.3	34.7
Total (%)	100.0	52.4	47.6
Metabolic risk	*n* = 1048^b^		
None (%)	14.8	8.4	91.6
Increased (%)	21.4	27.7	72.3
Substantially increased (%)	63.8	72.0	28.0
Total (%)	100.0	53.1	46.9

a n = 851 (81.0%) of the sample presented at least one of the pathologies.

b n of cases reported for the variable.

### Cognitive and mood dimensions

For the PCA, the following analysis steps were conducted: (1) the DSST data were not considered due to the small sample size that also included the measure together with no other missing value (*n* = 557), yielding a total of *n* = 684 subjects with no missing values considered, and (2) sequential exclusions of the parameters GDS, COWAT-FAS non-admissible and SRT intrusions and digit span forward, due to low component loadings (<0.400). From the analysis, the parameters GDS and DSST were considered single dimensions (termed “GDS” and “DSST”, respectively). The remaining parameters formed composites, termed: “GENEXEC” (general and executive function, Cronbach's alpha 0.793) composed of the parameters MMSE, Stroop (parameters: words, colors and words/colors), FAS (parameter: admissible) and digits (parameter: backward); and “MEM” (memory function, Cronbach's alpha 0.890) composed of the SRT test variables (parameters: CLTR, LTS, and delayed recall) (Table 2). Thus, a total of four dimensions were obtained and considered in the remaining analysis: GENEXEC, MEM, DSST, and GDS. The analysis followed and was in agreement with previously reported observations (Santos et al., 2013).

**Table 2:** **Principal component analysis with Varimax rotation: identification of cognitive composite dimensions**.

	**Communalities**	**Component**	
		**1**	**2**
zStroop words	0.683	0.813	0.150
zStroop colors	0.672	0.802	0.173
zCOWAT FAS Admissible	0.488	0.660	0.228
zStroop words/colors	0.427	0.648	0.083
zDigits backward	0.385	0.522	0.336
Zmmse	0.399	0.505	0.380
zSRT LTS	0.850	0.208	0.898
zSRT CLTR	0.841	0.198	0.896
zSRT delayed recall	0.746	0.221	0.835
Eigenvalue		2.818	2.674
% of variance (Cumulative %)		31.3	29.7 (61.0)
Cronbach's alpha		0.793	0.890

### Cognitive score

**Figure 1:**
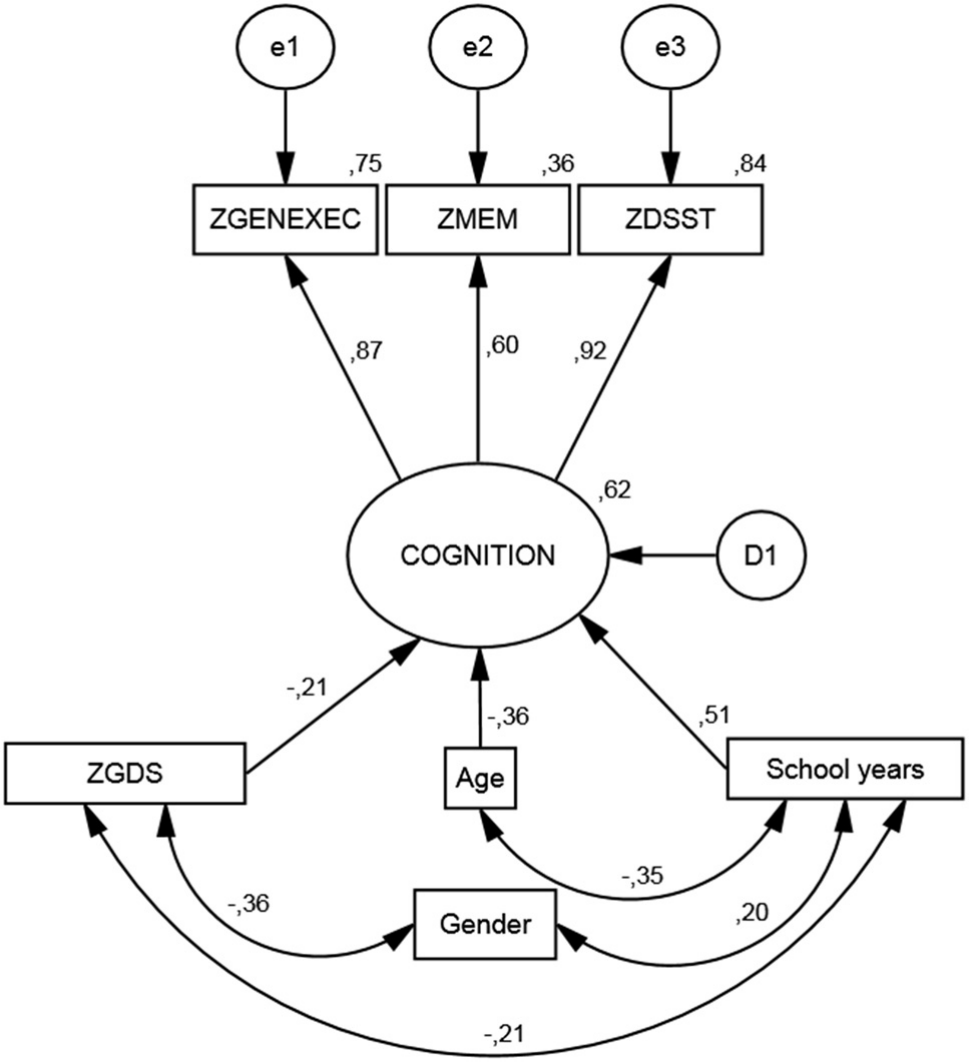
**Structural equation model.** Standardized coefficients for the cognition measurement model and its predictors.

The SEM analysis revealed a satisfactory fit level, χ^2^_(11)_ = 61.6; *p* < 0.001 |CFI = 0.974 |RMSEA (HI90) = 0.066 (0.083) (Figure 1). As it relates to the measurement model, the “latent” dimension of the modeling indicated significant coefficient regression weight for all cognitive dimensions (zGENEXEC, fixed at 1; zMEM, *z* = 19.6; zDSST, *z* = 29.3; for all *p* < 0.001). Regarding the structural model, the included variables explained 62% of the variability of the derived cognition score. The variable with the highest regression weight in explaining the cognition score was the number of school years (β schoolyears.cognition = 0.506; *z* = 18.4; *p* < 0.001); the higher the number of school years the higher the derived cognitive score. The second highest predictor was age (β age.cognition = −0.380; *z* = −14.6; *p* < 0.001), meaning that an increase in age was accompanied by a decrease in cognitive performance. The last direct predictor was the variable (depressive) mood (β zGDS.cognition = −0.207; *z* = −8.37; *p* < 0.001), which was also included as a dependent variable in the model. Gender was not significant in directly explaining the derived cognition score (β gender.cognition = 0.040; *p* = 0.121); therefore, the direct effect was not included in the final model. However, gender was significantly correlated with school years (*r* = 0.20; *p* < 0.001), where males tend to have more school years. Furthermore, gender negatively affected mood (*r* = −0.36; *p* < 0.001), meaning that males presented lower GDS scores. The zGDS was significantly predicted by gender and school years (16% of explained variance). A model including age as a predictor of zGDS was also conducted; however, this relation was non-significant (β age.zGDS = −0.010, *p* = 0.746). A negative and significant correlation was also observed between school years and age (*r* = −0.35; *p* < 0.001). The interaction between these two quantitative variables (age × school years) was tested (after centering each variable) with no significant results for any of the cognitive dimensions. Finally, school years negatively explained zGDS (*r* = −.21; *p* < 0.001); that is, with increasing school years, lower zGDS score were observed.

### The effect of socio-demographic, clinical, and lifestyle variables on cognitive and mood dimensions

**Figure 2:**
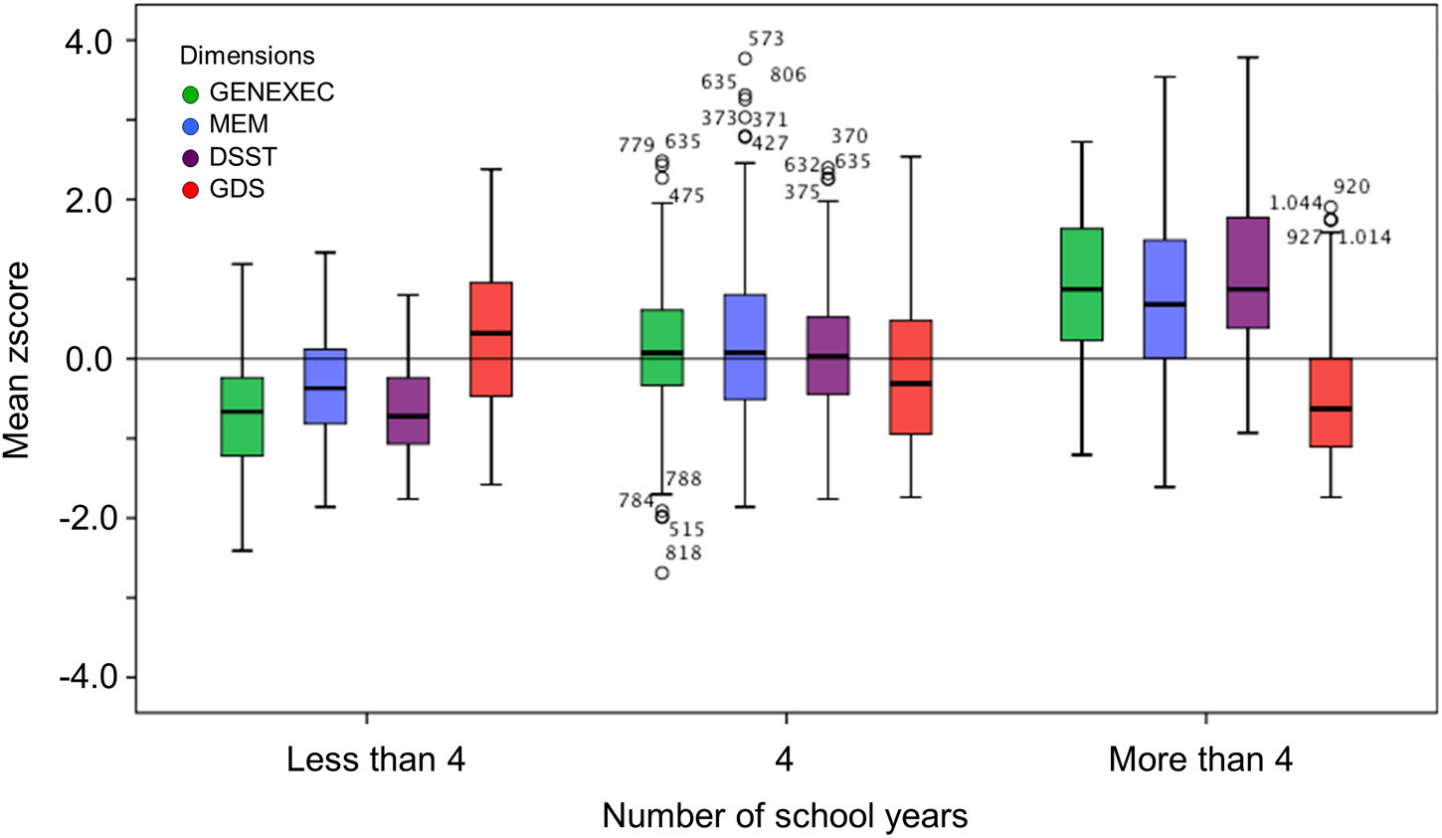
**Box-and-Whisker plots for cognitive and mood performance in the “more than 4,” “4,” and “less than 4” school years school groups.** First quartile, median, third quartile, interquartile range, outliers, and boundaries for outliers are represented.

**Figure 3:**
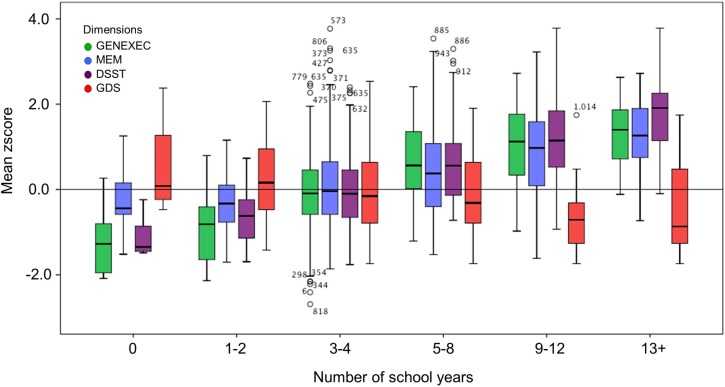
**Box-and-Whisker plots for cognitive and mood performance in the six school groups (0, 1–2, 3–4, 5–8, 9–12, and 13+ school years).** First quartile, median, third quartile, interquartile range, outliers, and boundaries for outliers) are represented.

The study sample was categorized by school years: “less than 4,” “4, completed primary education,” and “more than 4” (Table 3). The categorization was based on multiple observations. From the SEM analysis, education was the variable that explained the largest proportion of variance in the derived cognitive score (highest β value; Figure 1). The median number of school years for the cohort was 4 (primary school corresponded to the major educational barrier 50–60 years ago in Portugal), and previous findings indicated that there is a moderating effect of school years in the relation between age and mood (Santos et al., 2013). As such, the pattern of correlation between the cognitive dimensions was determined for the three educational groups considered. The higher the number of school years the higher the (significant) correlation between zGENEXEC with zMEM and zDSST; the same significant pattern was observed between zMEM and zDSST. Regarding zGDS, with an increase in school years the higher the negative correlation between zGDS and zGENEXEC and zMEM (although, it did not reach statistical significance for the “less than 4” school years group). For zDSST, a significant correlation with zGDS was only observed in the category “4” school years. The results across the cognitive and mood dimensions for each school group were visualized by boxplot (Figures 2, 3). Overall, cognitive measures increased with the number of school years, while depressed mood decreased.

**Table 3:** **Pearson correlation coefficients for cognition and mood dimensions**.

**School years**		**zGENEXEC**	**zMEM**	**zGDS**
Less than 4 years	zMEM	0.340^**^		
	zGDS	−0.154	−0.050	
	zDSST	0.614^**^	0.257^**^	−0.127
4 years	zMEM	0.355^**^		
	zGDS	−0.228^**^	−0.127^*^	
	zDSST	0.683^**^	0.390^**^	−0.211^**^
More than 4 years	zMEM	0.499^**^		
	zGDS	−0.342^**^	−0.242^**^	
	zDSST	0.705^**^	0.503^**^	−0.126

* p < 0.05 level;

** p < 0.01.

The educational groups categorization was maintained for the multiple hierarchical linear regressions (Tables 4A–C). For all school categories, and for all dimensions explored (dependent variables; zGENEXEC, zMEM, zDSST, and zGDS), the regression model was significant regarding the first regression block (age and gender variables). Specifically, total variance explained between 5.2% (adjusted *R*^2^, zDSST, “more than 4” school years) and 11.1% (adjusted *R*^2^, zDSST, “4” school years). Age was the most significant predictor in the block except for the zGDS dimension (in all school categories). Regarding gender, it only contributed significantly for zGENEXEC (β = 0.177, *p* < 0.001) and zDSST (β = 0.183, *p* < 0.01) in the “4” school years group and for zMEM (β = −0.114, *p* < 0.05) in the “less than 4” school years. The second regression analysis block (clinical variables and GDS score) was not significant in any of the dimensions considered in the “less than 4” school years group, meaning that these variables were not relevant in explaining cognitive performance and mood, controlling for age and gender. For the “4” and “more than 4” school years groups the second block [increment in explanation between 4.1% (Δ adjusted *R*^2^, zDSST, “4” school years) and 8.8% (Δ adjusted *R*^2^, zGENEXEC, “more than 4” school years)] contributed significantly to explain zGENEXEC, zMEM, and zDSST (the latter, only for the category “4” school years). The block was not significant in explaining zGDS. Furthermore, the variable GDS score was the only significant predictor in the second block variables. The significant regression weight varied between β = −0.219 (*p* < 0.001; zDSST, “4” school years) and β = −0.339 (*p* < 0.01, zGENEXEC, “more than 4” school years), meaning higher values in the GDS scale corresponded with lower performance in the cognitive dimensions. The third regression analysis block (lifestyle and physical parameter variables) was not significant in additionally explaining the zMEM and zDSST dimensions (in any of the school categories). For zGENEXEC the model was significant in the “less than 4” school years group (increment in explanation 4.2%, Δ adjusted *R*^2^) and for zGDS in the “less than 4” and “4” school years groups (respectively, increment in explanation 5.0% and 1.5%, Δ adjusted *R*^2^). Physical activity (β = 0.189, *p* < 0.05) and alcohol consumption “50 or less” (β = 0.154, *p* < 0.05) had a positive relation with zGENEXEC, which means it related with better performance in executive functions. Controlling for all variables a negative relation was observed between alcohol consumption and zGDS in both school groups; although, it should be noted that the “no alcohol consumption” group was composed of 77.9% females and that the “more than 50 gr/day” group was composed of 82.5% males. Finally, “BMI overweight” was negatively related with zGDS (β = −0.187, *p* < 0.05), thus revealing that moderate overweight (BMI [25.0–29.9]) was related with lower GDS and, indirectly, with better cognitive performance. The regression with the smallest sample size Table 4C) consisted of *n* = 122 participants, with an effect size of *R*^2^ = 0.156, a α error probability = 0.05 and 16 predictors, yielding an acceptable statistical power (1 - β error probability = 0.819).

**Table 4A:** **Hierarchical regression for variables predicting cognitive and mood dimension performance for individuals with less than 4 school years**.

		**zGENEXEC (*n* = 192)**			**zMEM (*n* = 333)**			**zDSST (*n* = 176)**			**zGDS (*n* = 342)**		
		**B [CI 95%]**	***SE***	**Beta**	**B [CI 95%]**	***SE***	**Beta**	**B [CI 95%]**	***SE***	**Beta**	**B [CI 95%]**	***SE***	**Beta**
**LESS THAN 4 SCHOOL YEARS**													
1	Gender^a^	−0.178[−0.412; 0.057]	0.119	−0.104	−0.188[−0.36; −0.015]	0.088	−0.114^*^	−0.096[−0.273; 0.08]	0.090	−0.077	−0.676[−0.893; −0.459]	0.110	−0.315^***^
	Age	−0.031[−0.047; −0.015]	0.008	−0.261^***^	−0.026[−0.038; −0.014]	0.006	−0.226^***^	−0.028[−0.04; −0.016]	0.006	−0.324^***^	−0.006[−0.021; 0.009]	0.008	−0.039
***R*^2^_adjusted_; *R*^2^; Δ*R*^2^**		0.071; 0.081; 0.081			0.06; 0.066; 0.066			0.103; 0.114; 0.114			0.097; 0.102; 0.102		
***F*_(*df*1, *df*2)_ for change in *R*^2^**		*F*_(2, 189)_ = 8.351^***^			*F*_(2, 330)_ = 11.688^***^			*F*_(2, 173)_ = 11.099^***^			*F*_(2, 339)_ = 19.301^***^		
2	Gender^a^	−0.223[−0.475; 0.028]	0.127	−0.131	−0.244[−0.433; −0.056]	0.096	−0.149^*^	−0.157[−0.353; 0.04]	0.099	−0.125	−0.729[−0.953; −0.504]	0.114	−0.34^***^
	Age	−0.029[−0.047; −0.012]	0.009	−0.248^***^	−0.025[−0.037; −0.012]	0.006	−0.217^***^	−0.027[−0.04; −0.014]	0.007	−0.313^***^	−0.009[−0.025; 0.006]	0.008	−0.062
	Stroke^b^	−0.076[−0.544; 0.391]	0.237	−0.023	−0.021[−0.284; 0.241]	0.133	−0.009	−0.195[−0.472; 0.082]	0.140	−0.102	0.169[−0.158; 0.496]	0.166	0.053
	Cardiac pathology^b^	−0.013[−0.293; 0.268]	0.142	−0.006	−0.109[−0.318; 0.1]	0.106	−0.056	0.091[−0.15; 0.332]	0.122	0.057	0.207[−0.058; 0.472]	0.135	0.081
	Diabetes^b^	0.033[−0.231; 0.296]	0.134	0.017	−0.012[−0.201; 0.176]	0.096	−0.007	0.081[−0.126; 0.287]	0.105	0.056	−0.066[−0.307; 0.176]	0.123	−0.028
	Dyslipidemia^b^	0.158[−0.058; 0.374]	0.109	0.102	0.069[−0.092; 0.231]	0.082	0.046	0.067[−0.104; 0.238]	0.087	0.056	−0.184[−0.387; 0.02]	0.103	−0.093
	Hypertension^b^	0.003[−0.225; 0.231]	0.115	0.002	−0.026[−0.196; 0.145]	0.087	−0.016	−0.088[−0.275; 0.098]	0.094	−0.070	−0.011[−0.227; 0.204]	0.110	−0.006
	GDS	−0.122[−0.24; −0.003]	0.060	−0.149^*^	−0.107[−0.192; −0.023]	0.043	−0.141^*^	−0.064[−0.159; 0.032]	0.048	−0.102	−	−	−
***R*^2^_adjusted_; *R*^2^; Δ*R*^2^**		0.077; 0.116; 0.035			0.069; 0.092; 0.026			0.105; 0.146; 0.033			0.102; 0.121; 0.018		
***F*_(*df*1, *df*2)_ for change in *R*^2^**		*F*_(6, 183)_ = 1.200			*F*_(6, 324)_ = 1.532			*F*_(6, 167)_ = 1.060			*F*_(5, 334)_ = 1.405		
3	Gender^a^	−0.117[−0.433; 0.198]	0.160	−0.069	−0.181[−0.413; 0.051]	0.118	−0.11	0.009[−0.235; 0.254]	0.124	0.008	−0.5[−0.781; −0.218]	0.143	−0.233^***^
	Age	−0.03[−0.047; −0.012]	0.009	−0.249^**^	−0.026[−0.039; −0.013]	0.006	−0.228^***^	−0.032[−0.046; −0.018]	0.007	−0.371^***^	−0.013[−0.029; 0.003]	0.008	−0.087
	Stroke	0.035[−0.444; 0.515]	0.243	0.011	−0.037[−0.303; 0.23]	0.136	−0.015	−0.171[−0.456; 0.115]	0.145	−0.089	0.085[−0.241; 0.41]	0.166	0.026
	Cardiac pathology	0.025[−0.262; 0.312]	0.145	0.012	−0.132[−0.345; 0.08]	0.108	−0.068	0.071[−0.175; 0.317]	0.125	0.044	0.19[−0.073; 0.453]	0.134	0.074
	Diabetes	0.002[−0.266; 0.269]	0.135	0.001	−0.032[−0.224; 0.16]	0.098	−0.018	0.029[−0.184; 0.243]	0.108	0.021	−0.124[−0.364; 0.116]	0.122	−0.052
	Dyslipidemia	0.15[−0.064; 0.364]	0.108	0.097	0.027[−0.137; 0.192]	0.083	0.018	0.056[−0.122; 0.234]	0.090	0.047	−0.131[−0.334; 0.072]	0.103	−0.066
	Hypertension	−0.027[−0.26; 0.206]	0.118	−0.017	−0.046[−0.22; 0.127]	0.088	−0.029	−0.069[−0.263; 0.125]	0.098	−0.055	0.004[−0.21; 0.219]	0.109	0.002
	GDS	−0.09[−0.208; 0.028]	0.060	−0.110	−0.09[−0.178; −0.002]	0.045	−0.117^*^	−0.06[−0.158; 0.038]	0.050	−0.096	−	−	−
	Alcohol 50 or less^c^	0.292[0.048; 0.535]	0.123	0.189^*^	0.128[−0.055; 0.311]	0.093	0.085	0.129[−0.076; 0.334]	0.104	0.108	−0.302[−0.525; −0.079]	0.113	−0.153^**^
	Alcohol more than 50^c^	0.178[−0.154; 0.509]	0.168	0.090	0.063[−0.188; 0.315]	0.128	0.034	−0.032[−0.304; 0.24]	0.138	−0.022	−0.561[−0.861; −0.26]	0.153	−0.229^***^
	Phy. act. less than 3^d^	0.299[−0.055; 0.653]	0.179	0.121	0.186[−0.085; 0.457]	0.138	0.074	0.045[−0.233; 0.323]	0.141	0.025	−0.147[−0.487; 0.192]	0.173	−0.044
	Phy. act. over 3^d^	0.346[0.03; 0.662]	0.160	0.154^*^	−0.118[−0.34; 0.105]	0.113	−0.057	−0.197[−0.461; 0.068]	0.134	−0.109	−0.188[−0.46; 0.084]	0.138	−0.07
	BMI overweight^e^	−0.125[−0.483; 0.234]	0.182	−0.081	0.179[−0.092; 0.45]	0.138	0.118	−0.057[−0.373; 0.258]	0.160	−0.048	−0.371[−0.703; −0.04]	0.169	−0.187^*^
	BMI obese^e^	−0.218[−0.628; 0.192]	0.208	−0.135	0.053[−0.248; 0.353]	0.153	0.034	−0.197[−0.546; 0.151]	0.177	−0.162	−0.076[−0.447; 0.294]	0.188	−0.038
	Met. risk increased^f^	0.099[−0.387; 0.585]	0.246	0.047	−0.157[−0.518; 0.204]	0.184	−0.070	0.137[−0.282; 0.557]	0.212	0.076	−0.051[−0.497; 0.395]	0.227	−0.018
	Met. risk subst. increased^f^	0.4[−0.125; 0.926]	0.266	0.222	0.087[−0.3; 0.473]	0.197	0.048	0.394[−0.067; 0.854]	0.233	0.267	0.073[−0.407; 0.552]	0.244	0.031
*****R***^2^_adjusted_; ***R***^2^; Δ*R*^2^**		0.119; 0.193; 0.077			0.08; 0.125; 0.033			0.113; 0.194; 0.048			0.152; 0.189; 0.069		
*****F***_(*df*1, *df*2)_ for change in *R*^2^**		*F*_(8, 175)_ = 2.089^*^			*F*_(8, 316)_ = 1.478			*F*_(8, 159)_ = 1.183			*F*_(8, 326)_ = 3.45^***^		

t-statistics:

* p < 0.05 level;

** p < 0.01;

*** p < 0.001.

a Gender, reference category: female;

b Pathology, reference category: no pathology;

c Alcohol measured in gr/day, reference category: none;

d Physical activity in number of times per week, reference category: none;

e BMI, reference category: underweight/normal;

f Metabolic risk, reference category: none.

**Table 4B:** **Hierarchical regression for variables predicting cognitive and mood dimension performance for individuals with 4 school years**.

		**zGENEXEC (*n* = 467)**			**zMEM (*n* = 483)**			**zDSST (*n* = 307)**			**zGDS (*n* = 487)**		
		**B [CI 95%]**	***SE***	**Beta**	**B [CI 95%]**	***SE***	**Beta**	**B [CI 95%]**	***SE***	**Beta**	**B [CI 95%]**	***SE***	**Beta**
**4 SCHOOL YEARS**													
1	Gender^a^	0.299[0.15; 0.448]	0.076	0.177^***^	−0.1[−0.27; 0.071]	0.087	−0.052	0.28[0.111; 0.448]	0.086	0.183^**^	−0.613[−0.785; −0.44]	0.088	−0.309^***^
	Age	−0.032[−0.04; −0.023]	0.004	−0.331^***^	−0.031[−0.041; −0.021]	0.005	−0.278^***^	−0.03[−0.04; −0.021]	0.005	−0.343^***^	−0.003[−0.013; 0.007]	0.005	−0.027
***R*^2^_adjusted_; *R*^2^; Δ*R*^2^**		0.11; 0.114; 0.114			0.083; 0.087; 0.087			0.111; 0.116; 0.116			0.096; 0.100; 0.100		
***F*_(*df*1, *df*2)_ for change in *R*^2^**		*F*_(2, 464)_ = 29.772^***^			*F*_(2, 480)_ = 22.764^***^			*F*_(2, 304)_ = 20.010^***^			*F*_(2, 484)_ = 26.824^***^		
2	Gender^a^	0.17[0.015; 0.324]	0.078	0.100^*^	−0.247[−0.423; −0.072]	0.089	−0.128^**^	0.186[0.011; 0.361]	0.089	0.122^*^	−0.629[−0.804; −0.455]	0.089	−0.317^***^
	Age	−0.031[−0.039; −0.022]	0.004	−0.318^***^	−0.032[−0.042; −0.022]	0.005	−0.287^***^	−0.03[−0.04; −0.02]	0.005	−0.340^***^	−0.006[−0.016; 0.004]	0.005	−0.052
	Stroke^b^	−0.017[−0.335; 0.302]	0.162	−0.005	0.022[−0.322; 0.366]	0.175	0.005	−0.029[−0.377; 0.319]	0.177	−0.009	0.429[0.069; 0.788]	0.183	0.103^*^
	Cardiac pathology^b^	−0.148[−0.38; 0.084]	0.118	−0.056	0.13[−0.131; 0.391]	0.133	0.043	−0.139[−0.393; 0.115]	0.129	−0.06	0.088[−0.185; 0.36]	0.139	0.029
	Diabetes^b^	0.001[−0.182; 0.184]	0.093	0.000	−0.198[−0.404; 0.007]	0.105	−0.082^#^	−0.187[−0.393; 0.02]	0.105	−0.096	0.082[−0.132; 0.296]	0.109	0.033
	Dyslipidemia^b^	0.034[−0.112; 0.18]	0.074	0.020	−0.014[−0.18; 0.152]	0.084	−0.007	0.082[−0.08; 0.244]	0.082	0.054	0.075[−0.098; 0.247]	0.088	0.037
	Hypertension^b^	−0.127[−0.273; 0.019]	0.074	−0.075	0.025[−0.142; 0.192]	0.085	0.013	−0.031[−0.195; 0.133]	0.083	−0.021	0.038[−0.137; 0.213]	0.089	0.019
	GDS	−0.215[−0.292; −0.139]	0.039	−0.252^***^	−0.246[−0.332; −0.16]	0.044	−0.252^***^	−0.17[−0.258; −0.083]	0.044	−0.219^***^	−	−	−
***R*^2^_adjusted_; *R*^2^; Δ*R*^2^**		0.168; 0.182; 0.068			0.138; 0.152; 0.066			0.152; 0.174; 0.058			0.103; 0.116; 0.016		
***F*_(*df*1, *df*2)_ for change in *R*^2^**		*F*_(6, 458)_ = 6.362^***^			*F*_(6, 474)_ = 6.112^***^			*F*_(6, 298)_ = 3.466^**^			*F*_(5, 479)_ = 1.722		
3	Gender^a^	0.155[−0.04; 0.349]	0.099	0.092	−0.234[−0.454; −0.013]	0.112	−0.121^*^	0.097[−0.129; 0.323]	0.115	0.064	−0.535[−0.76; −0.31]	0.115	−0.27^***^
	Age	−0.031[−0.039; −0.022]	0.004	−0.318^***^	−0.032[−0.042; −0.022]	0.005	−0.288^***^	−0.028[−0.039; −0.018]	0.005	−0.321^***^	−0.007[−0.017; 0.004]	0.005	−0.059
	Stroke	−0.003[−0.321; 0.314]	0.162	−0.001	0.017[−0.327; 0.36]	0.175	0.004	−0.006[−0.358; 0.347]	0.179	−0.002	0.412[0.055; 0.77]	0.182	0.099^*^
	Cardiac pathology	−0.181[−0.413; 0.051]	0.118	−0.068	0.128[−0.134; 0.39]	0.133	0.043	−0.172[−0.43; 0.086]	0.131	−0.074	0.093[−0.18; 0.365]	0.139	0.03
	Diabetes	−0.005[−0.192; 0.181]	0.095	−0.002	−0.191[−0.4; 0.019]	0.107	−0.079	−0.169[−0.382; 0.044]	0.108	−0.087	0.107[−0.11; 0.325]	0.111	0.043
	Dyslipidemia	0.029[−0.116; 0.174]	0.074	0.017	−0.017[−0.182; 0.149]	0.084	−0.009	0.078[−0.085; 0.241]	0.083	0.052	0.077[−0.094; 0.249]	0.087	0.039
	Hypertension	−0.129[−0.276; 0.019]	0.075	−0.076	0.033[−0.135; 0.201]	0.086	0.017	−0.022[−0.188; 0.144]	0.084	−0.014	0.017[−0.158; 0.193]	0.089	0.009
	GDS	−0.21[−0.287; −0.133]	0.039	−0.245^***^	−0.23[−0.318; −0.143]	0.044	−0.236^***^	−0.17[−0.259; −0.082]	0.045	−0.219^***^	−	−	−
	Alcohol 50 or less^c^	−0.047[−0.225; 0.13]	0.090	−0.028	0.173[−0.03; 0.376]	0.103	0.09	−0.06[−0.266; 0.146]	0.105	−0.04	−0.333[−0.542; −0.125]	0.106	−0.168^**^
	Alcohol more than 50^c^	0.002[−0.221; 0.224]	0.113	0.001	0.136[−0.118; 0.39]	0.129	0.061	−0.031[−0.28; 0.217]	0.126	−0.018	−0.27[−0.533; −0.007]	0.134	−0.118^*^
	Phy. act. less than 3^d^	0.231[0.036; 0.427]	0.099	0.103^*^	0.237[0.011; 0.462]	0.115	0.091^*^	0.098[−0.12; 0.315]	0.111	0.05	−0.155[−0.39; 0.079]	0.119	−0.058
	Phy. act. over 3^d^	−0.037[−0.211; 0.137]	0.089	−0.018	−0.147[−0.345; 0.05]	0.100	−0.065	−0.045[−0.249; 0.158]	0.103	−0.024	−0.134[−0.34; 0.072]	0.105	−0.057
	BMI overweight^e^	0.075[−0.126; 0.277]	0.102	0.045	−0.073[−0.301; 0.155]	0.116	−0.038	0.07[−0.158; 0.299]	0.116	0.046	−0.101[−0.338; 0.135]	0.120	−0.051
	BMI obese^e^	0.084[−0.156; 0.324]	0.122	0.046	−0.135[−0.407; 0.138]	0.139	−0.065	0.139[−0.138; 0.417]	0.141	0.082	0.049[−0.234; 0.333]	0.144	0.023
	Met. risk increased^f^	0.21[−0.033; 0.453]	0.124	0.105	−0.003[−0.278; 0.272]	0.140	−0.001	0.051[−0.224; 0.326]	0.140	0.029	0.025[−0.262; 0.312]	0.146	0.011
	Met. risk subst. increased^f^	0.029[−0.234; 0.291]	0.134	0.017	0.076[−0.224; 0.375]	0.152	0.039	−0.199[−0.506; 0.107]	0.156	−0.13	0.010[−0.303; 0.324]	0.160	0.005
***R*^2^_adjusted_; *R*^2^; Δ*R*^2^**		0.177; 0.205; 0.023			0.148; 0.176; 0.024			0.148; 0.193; 0.019			0.118; 0.145; 0.029		
***F*_(*df*1, *df*2)_ for change in *R*^2^**		*F*_(8, 450)_ = 1.636			*F*_(8, 466)_ = 1.680			*F*_(8, 290)_ = 0.844			*F*_(8, 471)_ = 2.028^*^		

t-statistics:

* p < 0.05 level;

** p < 0.01;

*** p < 0.001; ^#^p = 0.059.

a Gender, reference category: female;

b Pathology, reference category: no pathology;

c Alcohol measured in gr/day, reference category: none;

d Physical activity in number of times per week, reference category: none;

e BMI, reference category: underweight/normal;

f Metabolic risk, reference category: none.

**Table 4C:** **Hierarchical regression for variables predicting cognitive and mood dimension performance for individuals with over 4 school years**.

		**zGENEXEC (*n* = 156)**			**zMEM (*n* = 161)**			**zDSST (*n* = 122)**			**zGDS (*n* = 161)**		
		**B [CI 95%]**	***SE***	**Beta**	**B [CI 95%]**	***SE***	**Beta**	**B [CI 95%]**	***SE***	**Beta**	**B [CI 95%]**	***SE***	**Beta**
**MORE THAN 4 SCHOOL YEARS**													
1	Gender^a^	0.188[−0.129; 0.506]	0.161	0.092	0.047[−0.299; 0.393]	0.175	0.02	−0.084[−0.498; 0.33]	0.209	−0.036	−0.588[−0.882; −0.293]	0.149	−0.301^***^
	Age	−0.025[−0.04; −0.01]	0.008	−0.256^**^	−0.039[−0.055; −0.022]	0.008	−0.342^***^	−0.027[−0.046; −0.008]	0.010	−0.252^**^	0.002[−0.012; 0.016]	0.007	0.021
***R*^2^_adjusted_; *R*^2^; Δ*R*^2^**		0.057; 0.069; 0.069			0.105; 0.116; 0.116			0.052; 0.067; 0.067			0.078; 0.090; 0.090		
***F*_(*df*1, *df*2)_ for change in *R*^2^**		*F*_(2, 153)_ = 5.710^**^			*F*_(2, 158)_ = 10.350^***^			*F*_(2, 119)_ = 4.300^*^			*F*_(2, 158)_ = 7.785^***^		
2	Gender^a^	0.008[−0.321; 0.337]	0.166	0.004	−0.154[−0.52; 0.213]	0.186	−0.066	−0.094[−0.539; 0.352]	0.225	−0.04	−0.671[−0.968; −0.374]	0.150	−0.343^***^
	Age	−0.022[−0.037; −0.007]	0.008	−0.227^**^	−0.038[−0.055; −0.021]	0.009	−0.335^***^	−0.025[−0.045; −0.006]	0.010	−0.236^*^	0.002[−0.013; 0.017]	0.007	0.021
	Stroke^b^	−0.122[−0.89; 0.647]	0.389	−0.025	0.644[−0.218; 1.506]	0.436	0.113	−0.224[−1.735; 1.288]	0.763	−0.027	0.604[−0.132; 1.34]	0.373	0.126
	Cardiac pathology^b^	0.045[−0.389; 0.479]	0.220	0.016	−0.124[−0.61; 0.362]	0.246	−0.039	−0.033[−0.634; 0.569]	0.304	−0.01	0.468[0.055; 0.88]	0.209	0.174^*^
	Diabetes^b^	0.286[−0.128; 0.699]	0.209	0.106	0.002[−0.451; 0.456]	0.230	0.001	0.357[−0.174; 0.887]	0.268	0.124	0.177[−0.213; 0.567]	0.197	0.07
	Dyslipidemia^b^	−0.164[−0.459; 0.131]	0.149	−0.086	−0.078[−0.403; 0.248]	0.165	−0.036	−0.28[−0.669; 0.11]	0.197	−0.131	−0.19[−0.47; 0.089]	0.141	−0.105
	Hypertension^b^	−0.145[−0.452; 0.162]	0.155	−0.075	−0.104[−0.443; 0.236]	0.172	−0.048	−0.213[−0.619; 0.192]	0.205	−0.1	0.018[−0.274; 0.311]	0.148	0.01
	GDS	−0.355[−0.522; −0.189]	0.084	−0.339^***^	−0.351[−0.536; −0.165]	0.094	−0.294^***^	−0.178[−0.409; 0.053]	0.117	−0.144	−	−	−
***R*^2^_adjusted_; *R*^2^; Δ*R*^2^**		0.145; 0.189; 0.12			0.162; 0.203; 0.088			0.061; 0.123; 0.055			0.113; 0.152; 0.062		
***F*_(*df*1, *df*2)_ for change in *R*^2^**		*F*_(6, 147)_ = 3.610^**^			*F*_(6, 152)_ = 2.786^*^			*F*_(6, 113)_ = 1.191			*F*_(5, 153)_ = 2.238^#^		
3	Gender^a^	−0.072[−0.519; 0.375]	0.226	−0.035	−0.143[−0.64; 0.355]	0.252	−0.061	0.019[−0.601; 0.64]	0.313	0.008	−0.698[−1.11; −0.286]	0.208	−0.357^**^
	Age	−0.025[−0.04; −0.01]	0.008	−0.255^**^	−0.037[−0.054; −0.02]	0.009	−0.327^***^	−0.026[−0.046; −0.006]	0.010	−0.244^*^	0.002[−0.012; 0.017]	0.008	0.026
	Stroke	0.043[−0.738; 0.824]	0.395	0.009	0.759[−0.123; 1.642]	0.447	0.133	−0.142[−1.72; 1.436]	0.796	−0.017	0.585[−0.167; 1.337]	0.380	0.123
	Cardiac pathology	0.058[−0.385; 0.5]	0.224	0.021	−0.147[−0.647; 0.354]	0.253	−0.046	−0.064[−0.696; 0.569]	0.319	−0.02	0.533[0.112; 0.954]	0.213	0.199^*^
	Diabetes	0.315[−0.109; 0.738]	0.214	0.117	−0.009[−0.481; 0.464]	0.239	−0.003	0.365[−0.2; 0.931]	0.285	0.127	0.201[−0.203; 0.605]	0.204	0.079
	Dyslipidemia	−0.065[−0.374; 0.244]	0.156	−0.034	−0.035[−0.381; 0.311]	0.175	−0.016	−0.271[−0.686; 0.144]	0.210	−0.127	−0.151[−0.447; 0.145]	0.150	−0.083
	Hypertension	−0.158[−0.468; 0.151]	0.157	−0.082	−0.124[−0.47; 0.221]	0.175	−0.057	−0.188[−0.61; 0.234]	0.213	−0.087	0.002[−0.295; 0.299]	0.150	0.001
	GDS	−0.34[−0.511; −0.169]	0.087	−0.324^***^	−0.373[−0.564; −0.182]	0.097	−0.313^***^	−0.173[−0.42; 0.074]	0.125	−0.14	−	−	−
	Alcohol 50 or less^c^	0.174[−0.218; 0.566]	0.198	0.090	−0.034[−0.464; 0.396]	0.217	−0.016	−0.215[−0.777; 0.346]	0.283	−0.099	−0.345[−0.71; 0.019]	0.185	−0.188
	Alcohol more than 50^c^	0.097[−0.359; 0.554]	0.231	0.047	−0.212[−0.709; 0.284]	0.251	−0.091	−0.419[−1.052; 0.214]	0.319	−0.186	−0.164[−0.589; 0.261]	0.215	−0.083
	Phy. act. less than 3^d^	−0.004[−0.387; 0.379]	0.194	−0.002	0 [−0.424; 0.424]	0.215	0.000	0.075[−0.486; 0.635]	0.283	0.029	−0.035[−0.399; 0.329]	0.184	−0.016
	Phy. act. over 3^d^	−0.083[−0.431; 0.266]	0.176	−0.039	−0.362[−0.753; 0.03]	0.198	−0.150	0.122[−0.367; 0.611]	0.247	0.05	−0.293[−0.626; 0.039]	0.168	−0.146
	BMI overweight^e^	0.135[−0.217; 0.488]	0.178	0.071	0.364[−0.033; 0.761]	0.201	0.168	0.228[−0.252; 0.708]	0.242	0.107	0.112[−0.228; 0.453]	0.172	0.062
	BMI obese^e^	−0.54[−1.086; 0.006]	0.276	−0.197^#^	0.278[−0.332; 0.888]	0.309	0.09	0.007[−0.734; 0.748]	0.374	0.002	0.139[−0.384; 0.663]	0.265	0.054
	Met. risk increased^f^	−0.041[−0.449; 0.366]	0.206	−0.021	−0.002[−0.46; 0.457]	0.232	−0.001	−0.05[−0.631; 0.532]	0.293	−0.023	−0.089[−0.483; 0.304]	0.199	−0.048
	Met. risk subst. increased^f^	0.026[−0.482; 0.535]	0.257	0.013	−0.012[−0.58; 0.556]	0.287	−0.005	−0.111[−0.825; 0.603]	0.360	−0.05	−0.206[−0.692; 0.281]	0.246	−0.11
***R*^2^_adjusted_; *R*^2^; Δ*R*^2^**		0.157; 0.244; 0.055			0.158; 0.242; 0.039			0.027; 0.156; 0.033			0.114; 0.197; 0.046		
***F*_(*df*1, *df*2)_ for change in *R*^2^**		*F*_(8, 139)_ = 1.274			*F*_(8, 144)_ = 0.918			*F*_(8, 105)_ = 0.510			*F*_(8, 145)_ = 1.034		

t-statistics:

* p < 0.05 level;

** p < 0.01;

*** p < 0.001;

# p = 0.053.

a Gender, reference category: female;

b Pathology, reference category: no pathology;

c Alcohol measured in gr/day, reference category: none;

d Physical activity in number of times per week, reference category: none;

e BMI, reference category: underweight/normal;

f Metabolic risk, reference category: none.

To obtain final models with only significant predictors, four stepwise regressions were performed. Adding to the hierarchical model, the variable “BMI obese” was negatively related with zGENEXEC (β = −0.215, *p* < 0.01) in the “more than 4” school years group, which shows that more severe overweight (BMI [30.0+]) has a negative effect on cognitive (executive) performance. Finally, the clinical indices “stroke” (β = 0.104, *p* < 0.05, “4” school years) and “cardiac” (β = 0.207, *p* < 0.01, “more than 4” school years) significantly related with zGDS; that is, the presence of these pathologies corresponded to higher score in the GDS.

## Discussion

**Figure 4:**
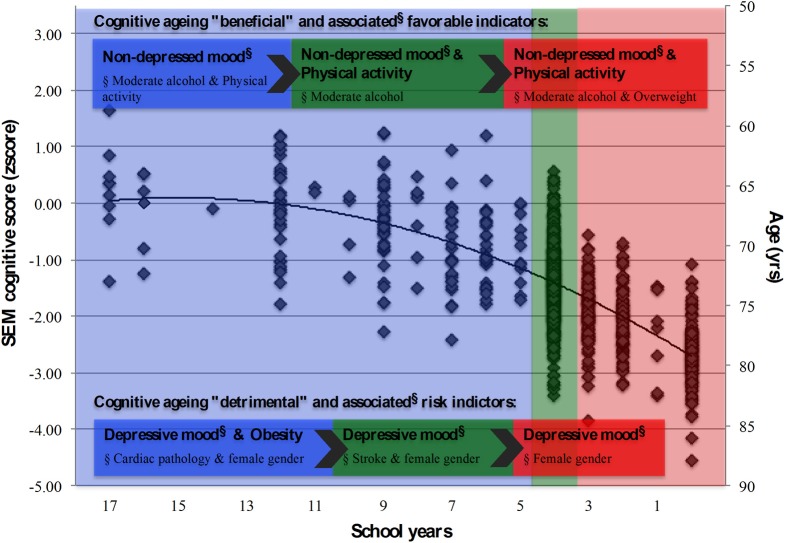
**Summary model for the latent cognition score (SEM) and associated structural and lifestyle, physical and clinical indicators.** School groups “more than 4,” “4,” and “less than 4” school years, represented in blue, green and red shaded areas, respectively. Cognitive “beneficial” and “detrimental” indicators, and their associated (beneficial/risk) factors (§), highlighted for each school group. Diamond symbols (◊) represent individuals.

Previously, we have reported on the positive association between education and cognitive performance in healthy aging, while age, female gender and, particularly, depressed mood were associated with poorer cognitive performances (Paulo et al., 2011; Santos et al., 2013). We now provide an integrative view of aging, aimed to characterize cognitive performance as it related with mood and clinical, physical and general lifestyle-related parameters (as possible “modifiable factors”) in distinct educational groups. We found that education was the main factor explaining the obtained cognitive score. Furthermore, the beneficial and risk factors associated with cognitive performance appeared to operate though mood, irrespectively of the number of school years (the main conclusions are illustrated in Figure 4).

### Cognition: age, education, and gender

Socio-demographic factors are important predictors of cognitive performance (Inouye et al., 1993) and not always through a direct pathway (Greendale et al., 2011; Ryba and Hopko, 2012). Foremost, not surprisingly, here, age was a common denominator explaining poorer cognitive performance. However, underlying the study rationale (division into school categories for exploration of other factors), the number of school years was the most important discriminatory parameter in explaining the derived cognitive performance. The results are not unexpected and follow other findings (for example, Ardila et al., 2000; Alberti et al., 2005; Wilson et al., 2009). One of the most well-established proxy measures of reserve capacity in the elderly is education, which is thought to promote more efficient cognitive processing or cognitive paradigms and use of brain networks, resulting in smaller cognitive declines (Stern, 2002, 2009). This indicates that learning increases the cognitive reserve and “protects” from age-related changes that can impair cognition, possibly due to an increase in neural connections made while learning (Vance et al., 2008). It may also mean that older individuals with greater experiential resources may exhibit better cognitive functioning and may have experienced a more continued engagement in problem solving-related activities (Vance et al., 2008; Zahodne et al., 2011). As such, this perspective may also underlie the present results indicating that more educated people relied less on memory strategies to solve problems when compared with less educated individuals (higher education level was accompanied with higher cognitive performance especially in executive and higher-level processing functions). However, future studies must necessarily address for this by assessing, for example, overall life experiences and attainments, including professional attainments, and for the presence (or not) of other sources of socio-cultural support/enrichment throughout childhood and adulthood, besides formal education. Notably, the analysis also revealed that the effect of gender on the cognitive score was indirectly mediated through education (females presented less school years) and mood (specifically, females presented higher GDS scores). While cultural aspects are of note in the association between female gender and less school years (until quite recently, in Portugal, males had a privileged access to school and other social environments), a more physiological aspect may underline the female gender/depressed mood relation (Nolen-Hoeksema, 2001; Greendale et al., 2011). More specifically, estrogen's effect on serotonergic function may be a key mechanism relating mood and cognition in the menopause. Estrogens have salutary neurophysiologic effects namely in the hippocampus and prefrontal cortex, which are rich in estrogen receptors and serve not only episodic and working memory but are also hypothesized to play a role in mood regulation (as reviewed, Greendale et al., 2011).

### Cognition: mood

Overall, results revealed that the higher the number of school years, the greater the effect of mood on cognition in both MEM (across all school categories) and GENEXEC/DSST (particularly in the higher educational levels) dimensions. Results are not entirely surprising from the statistical perspective: correlation values relate to the dispersion observed within variables. Here an increase in school years was related with an increase in dispersion in the cognitive dimensions. Furthermore, a “flooring effect” cannot be dismissed: individuals in the lower educational group are already in a lower cognitive class. Another consideration is that more educated individuals appear to show a resistance (or a more elevated threshold) for experiencing the (negative) cognitive effects of neuropathology factors (for example, Bennett et al., 2003; Dufouil et al., 2003). This can possibly be attributed to an enriched lifetime and current lifestyle environment that is reflected in brain reserve (Stern, 2002, 2009; Steffener and Stern, 2012). Also, studies report that depressive symptoms are associated with lower performance particularly in less-educated individuals (La Rue et al., 1986; Wight et al., 2002). On the other hand, more recent work, taking into consideration some limitations of other studies, has evidenced that brain reserve, as a proxy for educational level, does not mitigate cognitive decline associated with depression (Bhalla et al., 2005). Other factors that may be considered include: (i) our population sample is comprised of “healthy” agers without diagnosed neuropathology (although it cannot be dismissed that undiagnosed cases may have been present), (ii) it comprises a much larger sample, and (iii) mood is shown here to be a continuous variable rather than a systematic categorization. Altogether, it is here postulated that rather than a psychological element (for example, worse/better coping skills) a more complex neuroplasticity factor may underlie the apparent more “mood-sensitive” (despite presenting lower GDS scores) characteristics presented by stronger cognitive performers.

### Cognition: clinical, physical, and lifestyle indicators

Interestingly, the clinical and lifestyle variables effect was largely determined by mood. Specifically, for the clinical indices parameters those with cardiac or stroke pathologies presented higher GDS scores (more so in the two highest school categories). Perhaps surprisingly, the body of literature concurrently relating clinical parameters, education, mood and cognitive performance is scarce. Nonetheless, the methodology and results presented here support the negative influence of depression on verbal fluency performance independently of demographic variables or medical burden (Yochim et al., 2006). Furthermore, while cerebrovascular risk factors correlated with depressive symptoms, these independently did not predict verbal fluency (Yochim et al., 2006). This may indicate that factors that lead to depression can also lead to impairments on some cognitive domains, which is in line with our present findings and with the notion that there is a continuum between cognitive deficits and mood, especially during aging (Sotiropoulos et al., 2008). In respect to the lifestyle variables, the more significant results related with moderate increases in BMI, alcohol consumption and physical activity. Particularly, BMI appeared the offer a potential degree of “protection” for the less educated group. It is argued that body fat mass may exert some protection in older women and this effect may be mediated by endogenous estrogen produced by visceral adiposity (Bagger et al., 2004). This association, however, is not dose dependent since obesity seems to be detrimental. In fact, obesity can cause or exacerbate some diseases (Mikhail et al., 1999; Kopelman, 2000), and some of the co-morbidities that are related to obesity are well known to have a detrimental impact on cognitive performance ((Biessels et al., 2008; Novak and Hajjar, 2010), which also parallels our present results. Regarding alcohol consumption, the effects were largely observed in the lower school categories, where lower dose alcohol consumption appeared to actually have a beneficial effect in mood and cognition. The effect of alcohol on cognitive performance has been largely studied and the beneficial effects of consumption in opposite to abstinence or over-drinking are recognized (Ngandu et al., 2007; Chan et al., 2009; Arntzen et al., 2010; Zanjani et al., 2013), including light-to-moderate alcohol consumption being associated with a reduced risk of dementia (Ruitenberg et al., 2002). Here it would be further interesting to explore if findings related with specific categories of alcohol, instead of the total calculated gr/day. Also, a finer gradation would also be of key interest in light of the present findings; that is, more intervals to encompass for finer distinctions such as for “light drinkers” (i.e., 0–25 gr/day) and “heavy drinkers” (i.e., 75–100 gr/day). Although evidence does not indicate that the relation between alcohol and dementia varies by type of alcoholic beverage in individuals aged 55 years or older (Ruitenberg et al., 2002, data also points toward the association between light-to-moderate wine consumption (but not beer and spirits) with better performance on cognitive tests over time (Arntzen et al., 2010). Finally, here the results indicate that the lower the school category the more relevant the frequency of physical activity appears in particular in executive function. The beneficial effects of physical activity are well known in several domains of health status and aging (Vogel et al., 2009), including cognition (Hillman et al., 2008). This seems to be particularly noted for a positive association between physical activity and performance and executive level functions tests/tasks (Colcombe and Kramer, 2003; Ble et al., 2005; Eggermont et al., 2009). Interestingly, however, is the fact that the effect of physical activity on cognition can be not only direct but also operate through mood, agreeing with Vance et al. (2005). This suggests that the physical activity influence on cognition may operate by reducing depression/improving mood (Vance et al., 2005), and warrants in future studies further scrutiny by a more extensive characterization by type, intensity and duration of activity.

### Limitations and strengths of the study

Some study limitations and further directions should be addressed. Here we built in a hierarchical manner four models for the three separate levels of education. That is, three hierarchical models, one for each school group, for four outcomes (zGENEXEC, zMEM, zDSST, zGDS) and three blocks. The large number of models potentially increases the risk of a Type I error and may render results' interpretation difficult. This might have potential impact on statistical decision (reject or not reject the null hypothesis; statistical significance). To address any possible “false discovery,” several adjustments can be conducted (such as, Holm-Bonferroni, Holm-Sidak, and Benjamini-Hochberg). Here, our approach was, in parallel with p values, to also consider the practical significance (effect size), which according to Cohen (1988) is found to be “medium” at *R*^2^ > 0.13. Finally, also allowing for validation, we found variables (such as age) that statistically (and as expected) behave similarly across the defined groups. However, of note, particular care should be taken in the statistical power of the regression analyses for the higher educational group for the zDSST dimension given the small sample size for the number of variables considered. Therefore, in a similar integrated approach, further studies on the higher processing capacities of higher educated individuals, compared to lower educated ones, are warranted. Furthermore, here a puzzling finding was that (in general) an increase in school years related with an increase in dispersion in the cognitive dimensions; that is, individuals within the lower school years category presented a more homogeneous cognitive performance. The underlying reason may be one not here explored but that deserves a “second look” in further longitudinal assessments: that of lifetime intellectual engagement, including professional attainments/history and quality of life/social engagement (quantifiable via the use of validated scales/instruments).

From the findings, the clinical and lifestyle variables also require further methodological considerations. For instance, even if based on self-report with clinical history confirmation via medical records, patients with, for example, cerebral vascular disease, early mild cognitive impairment (MCI), or even preclinical AD, may have been putatively considered as part of the “normal aging” group. In the absence of throughout screening such as via structural imaging and/or biomarker studies, a precise/definitive diagnosis cannot take place and these were not necessarily present and/or were recent. Also, and perhaps a more realistic goal in medium to larger-scale studies, lifestyle variables such as physical activity and alcohol consumption should also be more extensively categorized. Additionally, a double verification should take place given that self-report may be unreliable. This would allow to more finely explore correlations between these and other variables of interest. Also, regarding the neurocognitive measures, the use of other general cognitive screening tools, such as the Montreal Cognitive Assessment (MoCA), could provide a more sensitive measure of performance over the MMSE, particularly if in the presence of MCI and/or cognitive decline (Lam et al., 2013). For a further integrated approach, three other considerations are also warranted: (i) confirmation of the self-reported disease status via measurable parameters; (ii) questionnaires on functional ability and dietary habits; and (iii) given the “governing” effect of mood, the pertinence to complement the present cohort studies with the evaluation of other mood dimensions such as stress and anxiety. Lastly, all measures were cross-sectional and, perhaps, mood in particular may be influenced by close events. Further longitudinal studies, which could address a more cause-effect relationship are needed.

Altogether upon considering the school categories separately and analyzing for the relevant variables in a hierarchical and step-wise manner, the results reveal that “depressive” mood emerged as a governing factor. The results obtained are vastly in line with, and expand on, relevant longitudinal and cross-sectional studies that consider associations between lifestyle and mood (van Gool et al., 2003, 2007), those that consider associations between social engagement, cognition and mood (Elwood et al., 1999; Gallacher et al., 1999), and those exploring education and mood (Bhalla et al., 2005). The work herein presented is novel in that for the same cohort, controlled for education, it explores several possible interactions between clinical/lifestyle, cognition and mood. Furthermore, by using SEM it was possible not only to measure a “cognition construct” but also to obtain indirect, direct and total effects of the structural variables. The findings are more so relevant from a clinical and practical point of view by, in combination with other studies, making possible to establish some recommendations that are associated with a better cognitive performance in older individuals, depending on the educational background.

## Author contributions

Nadine C. Santos and Patrício S. Costa performed the statistical and data analysis. Nadine C. Santos maintains the aging database. Pedro Cunha organized the evaluation sessions and participant recruitment and conducted the clinical characterization. Carlos Portugal-Nunes and Liliana Amorim contributed to lifestyle/physical and neurocognitive data analysis, respectively. Nadine C. Santos and Patrício S. Costa wrote the first draft of the manuscript. Nuno Sousa, Joana A. Palha, and Jorge Cotter conceived and designed the study. All authors participated in data collection and/or interpretation and contributed substantially to the scientific process leading up to the writing of the submitted manuscript, contributed to its writing and have approved of its final version.

### Conflict of interest statement

The authors declare that the research was conducted in the absence of any commercial or financial relationships that could be construed as a potential conflict of interest.
